# Genetic polymorphisms of Vascular Endothelial Growth Factor (VEGF) associated with endometriosis in Nigerian women

**DOI:** 10.1186/s40246-021-00364-x

**Published:** 2021-10-30

**Authors:** Ochuwa Adiketu Babah, Oyesola Oyewole Ojewunmi, Akinniyi Adediran Osuntoki, Melissa A. Simon, Bosede Bukola Afolabi

**Affiliations:** 1 Department of Obstetrics and Gynaecology, Faculty of Clinical Sciences, College of Medicine, University of Lagos/Lagos University Teaching Hospital, P.M.B. 12005, Idi-Araba, Surulere, Lagos, Nigeria; 2 DNA Laboratory, Sickle Cell Foundation Nigeria, Idi-Araba, Lagos, Nigeria; 3 School of Cancer and Pharmaceutical Sciences, King’s College London, Strand, London, WC2R 2LS UK; 4 Molecular Biology Unit, Department of Biochemistry, Faculty of Basic Medical Sciences, College of Medicine, University of Lagos, Lagos, Nigeria; 5 Department of Obstetrics and Gynecology, Feinberg School of Medicine, Northwestern University, 633 N St Clair, Suite 1800, Chicago, IL 60611 USA

**Keywords:** Endometriosis, Vascular endothelial growth factor, Ascites, Genetic polymorphism, Nigeria

## Abstract

**Objective:**

To determine if genetic polymorphism of VEGF is associated with the development of endometriosis in Nigerian women.

**Study design:**

Case control study of 100 women (50 healthy controls and 50 with endometriosis). Serum VEGF concentration of participants were determined using enzyme-linked immunosorbent assay (ELISA) technique. Genomic DNAs were isolated from peripheral blood samples and quantified by nanodrop spectrophotometer one. Single nucleotide polymorphisms genotyping was carried out by polymerase chain reaction and restriction fragment length polymorphism (PCR–RFLP).

**Results:**

Mean age of participants was 32.96 ± 6.91 years for control and 32.04 ± 7.56 years for cases. VEGF levels in case and control groups were not statistically different (82.68 pg/ml [69.11–121.11 pg/ml] vs. 82.81 pg/ml [72.90–113.82 pg/ml] respectively; *p* = 0.967). All four genotypes examined were in Hardy–Weinberg equilibrium. Minor allele frequency of − 460T > C, − 1154G > A, + 936C > T and + 2578C > A were 24%, 8%, 6% and 10% in the control and 19%, 9%, 5% and 14% in endometriosis patients. However, allele and genotype distributions of − 460T > C, − 1154G > A, + 936C > T and + 2578C > A VEGF polymorphisms in endometriosis patients and control were not significantly different (*p* > 0.05).

**Conclusion:**

Our preliminary findings revealed no association between endometriosis and − 460T > C, − 1154G > A, + 936C > T and + 2578C > A of VEGF genes among Nigerian women.

## Background

Endometriosis is defined as the presence of endometrial glands and stroma at sites other than the uterine cavity. In the general population, endometriosis affects 0.8–28.6% of women of reproductive age [1, 2]. It is a common cause of chronic pelvic pain, with a prevalence of 15.7% among infertile women undergoing laparoscopies [3]. The aetiology of endometriosis is unclear and many mechanisms have been postulated which include the coelomic metaplastic theory, implantation during surgical procedures and retrograde flow of menstruation [4]. The role of genetics in endometriosis has been studied over time and there appears to be a genetic predisposition in certain individuals by ancestry [5–7]. It has been found that African American women appear to have a predilection for uterine implants of endometriosis, and this presumably may be due to genetic or environmental differences [8].

Manifestation of endometriosis varies by populations; African women in particular present with a rare form, with ascites (often haemorrhagic) and peritonitis. Several cases have been reported in the literature [9–11] often in women of black African origin. These women are initially evaluated for ovarian malignancy and/or chronic liver or gastrointestinal pathology [10]. This delay in diagnosis and suspicion of a severe, chronic and possibly malignant condition is a cause of great anxiety and psychological trauma to the patients and their family. In a systematic review, it was found that endometriosis-related ascites usually affects non-White women of reproductive age; of the cases reported since 1954, 63% of those for whom ethnicity were specified were Africans [12]. Because of this unique disease manifestation, we hypothesized that a polymorphism of VEGF affects circulating VEGF levels and is associated with endometriosis and its varying presentations in Nigerian women.

Angiogenesis is known to be an essential part of endometriosis. VEGF is a homodimeric glycoprotein belonging to the growth factor family. It has vasculogenic and angiogenic properties and aids tissue proliferation. It plays a critical role in the proliferation of endothelial cell and participates in the development and progression of endometriotic lesions [13]. The mechanism by which it performs these functions is not clear. VEGF is encoded by a highly polymorphic VEGF gene mapped to chromosome 6p21.3, containing eight exons with a number of single nucleotide polymorphism (SNPs).

Genetic polymorphisms of VEGF have been reported in association with endometriosis in a number of studies [5, 14–17] but a few other studies did not establish such association [15, 18]. A higher incidence of VEGF + 405G > C gene was found in Turkish women with endometriosis compared to controls [16]. In another study, a significant difference was found in the genotype and allele distribution of VEGF + 405CC and + 936CC gene of women with endometriosis compared to controls [17]. No association was found between this VEGF gene and endometriosis in similar studies on Iranian women [15, 18]. Could this be because of racial differences? Five potentially functional SNPs (− 2578C > A, − 460T > C, − 1154G > A, + 405G > C and + 936C > T) of VEGF in the 3’-untranslated regions (UTRs) and the promoter region, impact significantly on VEGF plasma levels. Little is known about the polymorphisms of VEGF on the manifestation of endometriosis.

Significantly higher mRNA expression for VEGF concentrations has been reported in women with endometriosis compared to controls [19]. Various isoforms of VEGF, namely VEGFA, VEGF121, VEGF189, and VEGF111 have been found to be higher in peritoneal fluid during menstruation in women with endometriosis [20]. This study will explore the association between serum VEGF concentration and endometriosis and its association with genetic polymorphisms in VEGF gene.

Little improvement in the treatment modality has been achieved despite extensive research into the aetiology of endometriosis. Most studies on the genetics of endometriosis published in the literature were conducted in Western and Asian countries. Considering the scarcity of similar studies in Africa, taking into consideration racial and genetic differences in individuals of varying descent, we resolved to carry out this study on Nigerian women to establish if indeed there is a genetic variation in African women with endometriosis. This need for further studies on endometriosis in Africans or African Americans was emphasized in a study which reported an apparent predilection for uterine endometriotic implants in African American women, presumably due to genetic or environmental differences [8].

This study aimed at determining if there are genetic polymorphisms of VEGF associated with the development of endometriosis in Nigerian women, if there is a difference in the plasma VEGF levels in patients with endometriosis and women without endometriosis, and if there is a genetic variation (SNPs: − 460C > T, − 1154G > A, − 2578C > A and + 936C > T) in VEGF gene that is associated with endometriosis in Nigerian women.

## Methodology

### Study design and setting

This was a case–control study conducted between April 2018 and June 2020 at the Department of Obstetrics and Gynaecology of the College of Medicine of University of Lagos/ Lagos University Teaching Hospital (LUTH), Idi-Araba, Lagos, Nigeria.

### Study population

Consenting women with severe endometriosis (with and without ascites) as defined by the revised American Society of Reproductive Medicine Classification of Endometriosis [21] were recruited from the Gynaecology out-patient clinics, Accident and Emergency (A&E) unit and Gynaecological wards of the hospital as cases, while women who did not have endometriosis were recruited as controls. The cases included women diagnosed as having endometriosis either laparoscopically or at laparotomy, those who have been histologically confirmed as having endometriosis, or have shown clinical evidence of response to treatment for endometriosis. The controls were consenting women who have had laparoscopy or laparotomy for benign gynaecological conditions and confirmed as not having endometriosis. Excluded were women in whom diagnosis was unclear, women with adenomyosis or cancers, and women with chronic medical illness such as renal disease, cardiovascular disease, diabetes mellitus, or chronic infections.

### Sample size and sampling technique

A pilot study involving 50 women with endometriosis and 50 women without endometriosis was conducted to obtain preliminary data in Nigerian women. A convenience sampling technique was used. Participants were consecutively enrolled until the desired sample size was reached. For each case, the next available woman without endometriosis matched for age who consented to participate in the study was recruited as a control.

### Data collection

Prior to recruitment of eligible patients for this study, each patient had individual counselling during which the purpose of the study was explained, and informed written consent was obtained. Information was collected by interviewing the participants and from their case notes using the study proforma. The information collected included the patient’s age, parity, symptomatology, family history of endometriosis and any other genetic disorder in the family.

### Collection of specimens and handling

Four (4 ml) blood were collected by venepuncture from patients. Three millilitres of blood was transferred into a plain tube for serum and 1 ml was transferred into an EDTA tube for DNA extraction in the DNA laboratory of Sickle Cell Foundation Nigeria. Serial numbers were assigned to each specimen to conceal the identity of the patients (both cases and controls) from the laboratory scientists. Blood samples in plain tubes were placed on ice after collection and centrifuged at 3500 rpm for 10 min. The supernatants were stored at  − 80 °C until analysis. Blood samples in EDTA tube were kept at 2–8 °C and used for DNA extraction within 24 h.

### Laboratory analysis for serum VEGF concentration

Spectrophotometric determination of serum VEGF was done using Ray Biotech ELISA kits.

### DNA extraction

Genomic DNA was isolated from peripheral blood samples using spin column DNA extraction kit (Jena Bioscience, Germany) according to the manufacturer’s instructions**.** The DNA concentration was determined by a Nanodrop spectrophotometer One (Thermo Scientific, USA) and samples were stored at − 20 °C until analysis.

### Genetic analysis

SNPs genotyping was carried out using restriction fragment length polymorphism–polymerase chain reaction with published primers as described in earlier studies [13, 22, 23], 50 ng genomic DNA was amplified in a total reaction volume of 25 µl consisting 2.5 μl of 10X PCR buffer including Mgcl_2,_ 2.5 μl of 2 mM dNTPs, 1 μl of 10 μM each of forward and reverse primers, 0.2 μl (1U) Taq DNA Polymerase (Thermo Scientific, USA), and PCR grade water. List of primers, size of the amplicons, restriction enzymes, and the fragment sizes are as shown in Table 1.

**Table 1:** Restriction fragment length polymorphism conditions for VEGF SNPs

VEGF SNP	Primer sequence	Amplicon size	Restriction enzyme	Fragment lengths
− 460 T > C (rs833061)	TGTGCGTGTGGGGTTGAGCG (F) TACGTGCGGACAGGGCCTGA (R)	175 bp	Bsh1236I	CC: 155 and 20 bp; CT: 175, 155, and 20 bp; TT: 175 bp
−1154G > A (rs1570360)	TCCTGCTCCCTCCTCGCCAATG (F) GGCGGGGACAGGCGAGCATC (R)	206 bp	Mnl I	GG: 150 bp; GA:150, 34, 22 bp; AA: 184 and 22 bp
− 2578C > A (rs699947)	GGATGGGGCTGACTAGGTAAGC (F) AGCCCCCTTTTCCTCCAAC (R)	324 bp	Bgl II	CC: 324 bp; CA: 324, 202, and 122 bp; AA: 202 and 122 bp
+ 936C > T rs3025039	AAGGAAGAGGAGACTCTGCGC (F) TATGTGGGTGGGTGTGTCTACAG (R)	198 bp	Hsp92II	CC: 198 bp; CT: 198, 112 and 86 bp; TT: 112 and 86 bp

Polymerase chain reaction (PCR) was carried out in Applied Biosystems 9800 Fast thermocycler (Applied Biosystems, USA) under the following conditions: initial denaturation at 95 °C for 3 min, followed by 30 cycles of denaturation at 95 °C for 30 s, primer annealing at 60 °C for 30 s, extension at 72 °C for 90 s, and a final extension step of 72 °C for 2 min to terminate the process for − 460C > T; initial denaturation at 95 °C for 3 min, followed by 35 cycles of denaturation at 95 °C for 30 s, primer annealing at 65 °C for 30 s, extension at 72 °C for 1 min 50 s, and a final extension step of 72 °C for 2 min to terminate the process for − 1154 G > A; for − 2578 C > A: initial denaturation at 95 °C for 3 min, followed by 30 cycles of denaturation at 95 °C for 30 s, primer annealing at 58 °C for 30 s, extension at 72 °C for 1 min, and a final extension step of 72 °C for 2 min; for + 936 C > T, initial denaturation was at 95 °C for 3 min, followed by 30 cycles of denaturation at 95 °C for 30 s, primer annealing at 63 °C for 30 s, extension at 72 °C for 45 s, and a final extension step of 72 °C for 2 min.

Ten microliters PCR products were digested with 10U restriction enzymes (as shown in Table 1) in a total reaction volume of 20 μl following the restriction digest protocol in the manufacturer’s manual (New England Biolabs, Ipswich, MA, USA). All digested products were separated in 2.5% (w/v) agarose gel stained with ethidium bromide. Fifteen microliters of the digest and 5 μl bromophenol blue loading buffer were loaded in the agarose gel for electrophoresis and was visualized in a gel documentation system (Alpha Innotech, USA).

### Statistical analysis

Data obtained were presented as numbers, percentages, mean ± standard deviation. VEGF levels did not follow normal distribution and were presented as median [interquartile range]. Differences between continuous variables summarized as means were compared using independent student’s t test while VEGF levels were compared using Mann–Whitney U test. Frequencies of the patients with endometriosis and the controls were compared by Chi-square test (χ^2^) or linear-by-linear association. The odd ratios (OR) and their 95% confidence intervals (95%CI) were also calculated. All statistical analyses were carried out using SPSS version 25 for Windows (IBM Corp. Released 2017. IBM SPSS Statistics for Windows, Version 25.0. Armonk, NY: IBM Corp.) with *p*-value < 0.05 considered significant.

## Results

The difference in the mean age of participants in the control and case groups (32.96 ± 6.91 years vs. 32.04 ± 7.56 years) was not significant (*p* = 0.527). BMI was also not significantly different between the controls and cases (*p* = 0.790). Median parity in women with endometriosis (cases) was 0 (range 0–3) and in control groups was 0 (range 0–4), with 94% of cases and 60% of controls being nulliparous. Among the cases, chronic pelvic pain, painful menstruation, painful intercourse, painful urination, and painful defaecation were all significantly associated with endometriosis as expected (*p* < 0.0001). Also, abdominal swelling, cyclical bleeding from the umbilicus, ascites, and pleural effusion were associated with endometriosis (*p* < 0.05). Seventeen (34%) and seven (14%) participants with endometriosis had ascites and pleural effusion, respectively. Twenty-seven (54%) of patients had pelvic endometriosis, while 23 (46%) had extra-pelvic endometriosis. The difference between median serum VEGF concentration of both cases and controls were not significantly different (*p* = 0.967).The median serum VEGF concentration in women with histologically confirmed endometriosis versus those diagnosed by other methods did not show statistically significant difference compared to controls (*p* = 0.893) (Table 2).

**Table 2:** Demographics and characteristics of study participants

Characteristics	Control	Case	*p*-value
Age	32.96 ± 6.91	32.04 ± 7.56	0.527
BMI	24.97 ± 4.71	24.73 ± 4.18	0.790
Parity	0 (0–4)	0 (0–3)	< 0.0001
*Chronic pelvic pain*			
No	42 (84)	15 (30)	< 0.0001
Yes	8 (16)	35 (70)	
*Painful menstruation*			
No	26 (52)	1 (2)	< 0.0001
Yes	24 (48)	49 (98)	
*Painful intercourse*			
No	47 (94)	25 (50)	< 0.0001
Yes	3 (6)	22 (44)	
I don’t know	–	3 (6)	
*Bleeding/site of bleeding*			
None	50 (100)	40 (80)	0.001
Umbilicus	–	10 (20)	
*Blood in stool*			
No	49 (98)	47 (94)	0.702
Yes	–	3 (6)	
I don’t know	1 (2)	–	
*Painful defaecation*			
No	50 (100)	35 (70)	< 0.0001
Yes	–	15 (30)	
*Blood in urine*			
No	49 (98)	47 (94)	0.399
Yes	–	1 (2)	
I don’t know	1 (2)	2 (4)	
*Painful urination*			
No	50 (100)	36 (72)	< 0.0001
Yes	–	14 (28)	
*Convulsion during menstruation*			
No	50 (100)	49 (98)	0.317
I don’t know	–	1 (2)	
*Coughing up blood*			
No	50 (100)	50 (100)	-
*Abdominal swelling*			
No	49 (98)	35 (70)	< 0.0001
Yes	1 (2)	15 (30)	
*Ascites*			
No	50 (100)	33 (66)	< 0.0001
Yes	–	17 (34)	
*Pleural effusion*			
No	50 (100)	43 (86)	0.006
Yes	–	7 (14)	
*Type of endometriosis*			
Pelvic	50 (100)	27 (54)	< 0.0001
Extra-pelvic	–	23 (46)	
*No Symptom*			
NO	31 (62)	50 (100)	< 0.0001
YES	19 (38)	-	
*Family history of endometriosis*			
No	50 (100)	46 (92)	0.056
Yes	–	3 (6)	
I don’t know	–	1 (2)	
*Method of diagnosis*			
Laparotomy	47 (94)	19 (38)	< 0.0001
Laparoscopy	3 (6)	15 (30)	
Clinical features	–	14 (28)	
Histology	–	2 (4)	
Serum VEGF level (pg/ml)	82.81 [72.90 – 113.82]	82.68 [69.11 – 121.11]	0.967
*Serum VEGF level (pg/ml) based on methods of diagnosis*			
Histologically confirmed Non-histologic diagnosis	82.81 [72.90–113.82] – –	– 76.7 [67–123] 82.8 [70.8–113.6]	0.893

Age and BMI are presented as Mean (S.D.), Parity is presented as median (range), Serum VEGF level as Median (IQR), and others as frequency (percentage)

**Fig. 1:**
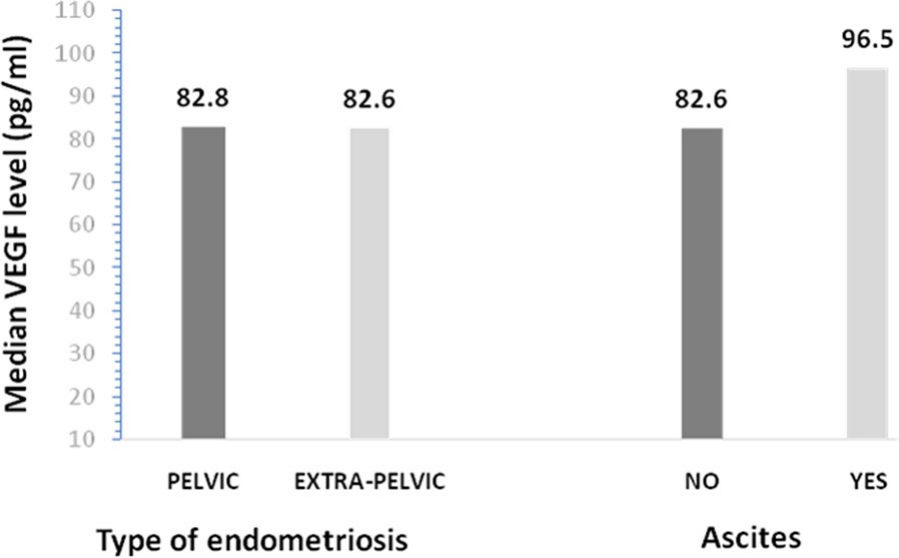
Serum VEGF concentrations in patients with endometriosis

Endometriosis patients with ascites had higher median serum VEGF concentration compared to those without ascites, but this was not statistically significant (*p* = 0.735). The concentrations of serum VEGF in patients with pelvic endometriosis was also not significantly different from those with extra-pelvic endometriosis (*p* = 0.471) (Fig. 1).

The minor allele frequencies of the VEGF SNPs investigated were compared between the control and case groups and the following were obtained—VEGF 936 C > T: 6% vs. 5% [odds ratio (OR) 0.82, 95% confidence interval (CI) 0.24–0.79], 460 T > C: 24% vs. 19%; (OR 0.67, 95%CI 0.34–1.31, 1154 G > A: 8% vs. 9% (OR 1.14, 95%CI 0.42–3.08), and 2578 C > A: 10% vs. 14% (OR 1.47, 95%CI 0.62–3.48). The genotype and allele frequencies of VEGF SNPs investigated were not statistically significant between the control and case groups (p > 0.05). All the four SNPs examined were all in Hardy–Weinberg equilibrium (p > 0.05) (Table 3).

**Table 3:** Genotype and allele frequencies of VEGF SNPs in control and endometriosis patients

	Control	Case	OR (95% CI); *p*-value
* + 936 C > T*			
CC	44 (88)	45 (90)	*p* = 0.749
CT	6 (12)	5 (10)	
HWE	0.652	0.709	
*Allele*			
C	0.94	0.95	0.82 (0.24–2.79)
T	0.06	0.05	*p* = 0.757
*−460T > C*			
TT	25 (50)	31 (82)	*p* = 0.178
TC	24 (48)	19 (38)	
CC	1 (2)	–	
HWE	0.080	0.097	
T	0.74	0.81	0.67 (0.34–1.31)
C	0.26	0.19	*p* = 0.237
*−1154 G > A*			
GG	42 (84)	41 (82)	*p* = 0.790
GA	8 (16)	9 (18)	
HWE	0.539	0.484	
G	0.92	0.91	1.14 (0.42–3.08)
A	0.08	0.09	*p* = 0.799
*−2578 C > A*			
CC	40 (80)	36 (72)	*p* = 0.349
CA	10 (20)	14 (28)	
HWE	0.432	0.249	
C	0.90	0.86	1.47 (0.62–3.48)
A	0.10	0.14	*p* = 0.386

In Table 4, we compared our results with other published reports comparing patients with endometriosis with controls. The minor allele frequencies reported for the SNPs in this study are much lower (except − 460T > C) than the published reports. However, they are similar to the minor allele frequencies of Yoruba Ibadan in Nigeria in 1000 genome reference; this is not surprising since most of the study participants are of Yoruba ethnic group.

**Table 4:** Comparison of VEGF polymorphisms in our study and the published reports

SNP	Population	Cases/controls	MAF [cases/controls]	*p*-value	References
+ 936 C > T rs3025039	Chinese	344/360	0.17/0.17	0.75	[13]
	Brazilians	165/95	0.15/0.15	0.63	[14]
	Iranians	100/200	0.1/0.11	0.5	[24]
	Japanese	147/181	0.27/0.20	0.05	[25]
	Tunisians	105/150	0.25/0.13	0.001	[26]
	This study	50/50	0.05/0.06	0.76	–
	1000 Genomes phase 3 (MAF): YRI—0.069; CEU—0.152; CHB—0.184; JPT—0.159 (27)				
−460T > C rs833061	Chinese	344/360	0.23/0.21	0.33	[13]
	Brazilians	179/107	0.43/0.40	0.50	[14]
	Japanese	147/181	0.28/0.33	0.23	[25]
	Tunisians	105/150	0.40/0.42	0.69	[26]
	This study	50/50	0.19/0.26	0.24	-
	1000 Genomes phase 3 (MAF): YRI—0.296; CEU—0.449; CHB—0.272; JPT—0.337 (27)				
−1154 G > A rs1570360	Chinese	344/360	0.16/0.22	0.004	[13]
	Brazilians	267/207	0.24/0.16	0.01	[28]
	This study	50/50	0.09/0.08	0.80	–
	1000 Genomes phase 3 (MAF): YRI—0.032; CEU—0.283; CHB—0.175; JPT—0.168 (27)				
−2578 C > A rs699947	Chinese	344/360	0.19/0.26	0.002	[13]
	Iranians	100/200	0.40/0.43	0.7	[24]
	Brazilians	218/288	0.40/0.32	0.03	[27]
	This study	50/50	0.14/0.10	0.39	-
	1000 Genomes phase 3 (MAF): YRI—0.12; CEU—0.455; CHB—0.272; JPT—0.337 (27)				

MAF: minor allele frequency; YRI: Yoruba in Ibadan, Nigeria; CEU: Utah residents with Northern and Western European ancestry; CHB: Han Chinese in Beijing, China; JPT: Japanese in Tokyo, Japan

The association between serum VEGF concentrations and VEGF SNPs did not show statistical significance (*p* > 0.05) (Table 5).

**Table 5:** Distribution of the VEGF SNPs according to VEGF levels

	Control	Case
+ 936 C > T	VEGF (pg/ml)	VEGF (pg/ml)
CC	84.62 [71.26–113.91]	82.59 [68.38–121.58]
CT	80.96 [73.18–102.38]	82.76 [71.68–111.21]
*p*-value	0.873	0.875
*−460T > C*		
TT	88.21 [71.51–116.16]	89.67 [68.94–118.39]
TC	80.79 [71.71–112.21]	77.97 [70.94–122.51]
CC	62.49	–
*p*-value	0.421	0.976
*−1154 G > A*		
GG	84.62 [70.52–109.15]	86.19 [70.72–122.99]
GA	79.29 [76.27–122.82]	71.00 [62.98–102.35]
*p*-value	0.576	0.096
*−2578 C > A*		
CC	83.67 [71.08–117.27]	82.68 [68.99–120.08]
CA	82.81 [75.35–109.09]	82.41 [71.73–123.96]
*p*-value	0.952	0.666

A comparison of genotype and allele frequencies of VEGF SNPs in controls versus endometriosis patients with ascites did not show statistically significant differences; the minor allele frequency of − 460T > C, − 1154G > A, + 936C > T and + 2578C > A were 24%, 8%, 6% and 10% in the control group and 5%, 5%, 1% and 4% in endometriosis patients with ascites, respectively (*p* > 0.05). There were no statistically significant differences found when the genotype and allele frequencies of VEGF SNPs were compared in controls versus endometriosis women with histological confirmation, and none in endometriosis women with histological versus non-histological diagnosis. Minor allele frequency of − 460T > C, − 1154G > A, + 936C > T and + 2578C > A was 24%, 8%, 6% and 10% in control versus 9%, 26%, 1% and 7% in endometriosis patients with histological confirmation, respectively (*p* > 0.05); and 9%, 2%, 1%, 7% versus 10%, 7%, 4%, 7% in endometriosis patients with histological versus non-histological diagnosis, respectively (*p* > 0.05).

Genotype distribution of VEGF SNPs examined did not differ significantly whether the endometriosis patients had ascites or not (Table 6). Also, the genotype distribution did not significantly influence the type of endometriosis (*p* > 0.05) (Table 7).

**Table 6:** Genotype frequencies of VEGF SNPs in endometriosis patients with and without ascites

	Ascites		OR (95% CI); *p*-value
	No	Yes	
+ 936 C > T Genotypes			
CC	29 (87.9)	16 (94.1)	0.45 (0.05–4.41) *p* = 0.490
CT	4 (12.1)	1 (5.9)	
−460T > C genotypes			
TT	19 (57.6)	12 (70.6)	0.57 (0.16–1.98) *p* = 0.369
TC	14 (42.4)	5 (29.4)	
−1154 G > A genotypes			
GG	29 (87.9)	12 (70.6)	3.02 (0.69–13.23) *p* = 0.136
GA	4 (12.1)	5(29.4)	
−2578 C > A genotypes			
CC	23 (69.7)	13 (76.5)	0.71(0.19–2.71) *p* = 0.617
CA	10 (30.3)	4(23.5)

**Table 7:** Genotype frequencies of VEGF SNPs endometriosis patients according to the endometriosis type

	Type of endometriosis		OR (95% CI); *p*-value
	Pelvic (N = 27) n (%)	Extra-pelvic (N = 23) n (%)	
+ 936 C > T			
Genotypes			
CC	24 (88.9)	21 (91.3)	0.76 (0.12–5.00)
CT	3 (11.1)	2 (8.7)	*p* = 0.779
−460 T > C			
Genotypes			
TT	14 (51.9)	17 (73.9)	0.38 (0.12–1.26)
TC	13 (48.1)	6 (26.1)	*p* = 0.109
−1154 G > A			
Genotypes			
GG	23 (85.2)	18 (78.3)	1.60 (0.37–6.83)
GA	4 (14.8)	5 (21.7)	*p* = 0.525
−2578 C > A			
Genotypes			
CC	18 (66.7)	18 (78.3)	0.56 (0.16–1.99)
CA	9 (33.3)	5 (21.7)	*p* = 0.363

## Discussion

Prior studies conducted in non-African settings that evaluated the role of VEGF in development of endometriosis have yielded conflicting results. Hence, this study investigated occurrence of endometriosis and genetic polymorphisms of VEGF 936 C > T, 460 T > C, 1154 G > A, and 2578 C > A in Nigerian women with endometriosis.

All the patients with endometriosis in this study had at least one symptom and a good proportion of them were recruited from the gynaecology oncology clinic, most having been referred to Lagos University Teaching Hospital due to suspicion of ovarian malignancy from abdominal distension due to ascites. This probably explains the high incidence of extrapelvic endometriosis especially the peritoneal type in this study. At surgery, these women with peritoneal endometriosis were found to have haemorrhagic ascites with cytology smears often showing inflammatory cells.

As previously established, black Nigerian women with endometriosis also have significantly lower parity (most being nulliparous) compared to those without endometriosis because of the association of endometriosis with infertility. Dysmenorrhoea was the commonest symptom reported in this study by all the women with endometriosis except one, followed by chronic pelvic pain. The possibility of heredity being a predisposing factor to the development of endometriosis was not established in this study as only 6% of affected women reported a history of endometriosis in a first degree relative. The availability of laparoscopy in our centre has improved our diagnosis rate and many women are often promptly diagnosed; the greater proportion of women in this study was diagnosed at laparotomy or laparoscopy.

Contrary to our findings, a significantly higher plasma VEGF concentration has been reported in Belgian and Tunisian (North-African) women with endometriosis compared to controls [26, 28]. In a study conducted in Romania, a lower serum VEGF concentration was found in women with endometriosis compared to apparently healthy controls, and these controls were selected based on findings from clinical assessment alone [29]. This is a limitation in their study as some women with mild endometriosis who were apparently healthy might have been recruited inadvertently as controls, thereby increasing selection bias. As was done in our study, some earlier studies recruited women who did not have endometriosis at laparoscopy or laparotomy as controls [28, 30]. We observed a high incidence of peritoneal endometriosis complicated by ascites, affecting one-third of Nigerian women with endometriosis who participated in this study. This is in support of earlier findings in which Black women were more likely to have ascites than the White women [9–12]. We also found the median serum VEGF level to be non-significantly higher in women with endometriosis with ascites (peritoneal endometriosis) compared to those without ascites. A significantly higher concentration of VEGF in peritoneal fluid of Iranian women with endometriosis compared to controls had earlier been reported [30]. The lack of statistical significance in our study may be due to the small sample size which is a major limitation in drawing conclusions from our findings. An appropriately powered study will help clarify this association. The higher VEGF concentration in the peritoneal fluid could be an adaptive mechanism facilitating the development of ectopic endometrial tissue in the peritoneum as macrophages in peritoneal fluid have been found to produce high amounts of VEGF [31]. This mechanism may explain why there is a higher serum level in those with ascites as against those without ascites. It has also been reported that peritoneal fluid VEGF concentration in women with endometriosis correlated with the stage of disease [32]. We did not explore this association between peritoneal fluid VEGF concentration and disease severity in our study due to paucity of patients with mild endometriosis.

As in this study, an earlier study in Iran reported no significant difference in the allele distribution of 936 C > T and 2578 C > A polymorphisms in 100 women with histologically confirmed endometriosis compared to 200 controls [24]. It has also been reported that no significant difference exists in the frequency and genotype distribution of VEGF 460T > C polymorphisms in 147 Japanese women with surgically diagnosed endometriosis compared to the 181 control group [25]. These studies were conducted in a non-African population and used a larger sample size compared to our study.

A meta-analysis found no association between 460 T > C, 1154 G > A, and 2578 C > A polymorphisms and occurrence of endometriosis [33]. Contrary to our findings, the meta-analysis found VEGF gene 936C > T polymorphism to be associated with an increased risk of endometriosis. In another study, it was found that a similar association exists between VEGF gene 936C > T polymorphism and women with severe (stages III and IV) endometriosis, an association that was lost when the gene polymorphism was compared in the entire Japanese population of endometriosis patients studied [25, 33]. The similarity in the serum VEGF level and VEGF genes (936 C > T, 460 T > C, 1154 G > A, and 2578 C > A) in pelvic and extrapelvic endometriosis suggests that VEGF alone is not adequate in explaining the pathogenesis of extrapelvic endometriosis. It is difficult to explain why there is disparity in the findings of these studies, but the method of diagnosing endometriosis and the diverse genetic background of the studied populations may be contributory. It is worthy of note that the genotype and allele frequencies in our study population are very low (this is also confirmed by the 1000 genome reference) [34] compared to other populations and larger sample size may be necessary to detect any significant effect, hence, our findings cannot be generalised yet.

Of interest is the higher incidence of peritoneal endometriosis in Nigerian women, which often is a cause of great concern to both patients and physicians due to fear of malignancy, and its association with a higher serum VEGF level in this study. Larger studies exploring VEGF and other relevant genes in Nigerian women, with and without peritoneal endometriosis, will be desirable.

## Conclusion

Women with ascites complicating endometriosis had higher non-statistically significant serum VEGF concentration, and we reported lower minor allele frequencies of VEGF SNPs compared to other populations, with no association between endometriosis and − 460T > C, − 1154G > A, + 936C > T and + 2578C > A of VEGF gene among Nigerian women.

## Data Availability

The datasets generated and analysed during the current study are not publicly available but are available from the corresponding author on reasonable request.
